# Carotid-Femoral Pulse Wave Velocity in Children in South Africa: Reference Values for the Vicorder Device

**DOI:** 10.1177/00033197251314218

**Published:** 2025-01-23

**Authors:** Claire Davies, Florin Vaida, Kennedy Otwombe, Mark F. Cotton, Sara Browne, Steve Innes

**Affiliations:** 1 Division of Epidemiology and Biostatistics, Faculty of Medicine and Health Sciences, Stellenbosch University, Stellenbosch, South Africa; 2 Division of Biostatistics and Bioinformatics, School of Public Health, University of California, San Diego, La Jolla, CA, USA; 3 Perinatal HIV Research Unit, Faculty of Health Sciences, University of the Witwatersrand, Johannesburg, South Africa; 4 School of Public Health, Faculty of Health Sciences, University of the Witwatersrand, Johannesburg, South Africa; 5 Family Center for Research with Ubuntu, Department of Paediatrics and Child Health, Stellenbosch University, Stellenbosch, South Africa; 6 School of Public Health, University of California, San Diego, La Jolla, CA, USA; 7 Desmond Tutu HIV Centre, University of Cape Town, Cape Town, South Africa

**Keywords:** children, pulse wave velocity, PWV, cardiovascular risk, reference values

## Abstract

Atherosclerosis often starts in childhood, tracking to adulthood. In children, early vascular disease can be detected as arterial stiffness. Carotid-femoral pulse wave velocity is considered the non-invasive gold standard method for measuring arterial stiffness and widely accepted for use in children. We define pulse wave velocity (PWV) reference values for African children, in a cohort of children and adolescents living in Cape Town, South Africa, using the oscillometric Vicorder device, and considering the anatomical pathway in growing children. Three hundred and twenty four children (6–16 years old) were followed annually at Tygerberg Hospital, from March 2014 to March 2020, yielding 959 longitudinal PWV measurements. Centile curves for males and females by age and height were constructed using the Lamda-Mu-Sigma (LMS) method. Our study demonstrates that African children have a relatively flat PWV throughout childhood and early adolescence, from 7 to 14 years of age, and between 120 and 170 cm standing height. These gender-specific percentiles for age and height will allow accurate surveillance of arterial elasticity in African children over time. The identification of children at high risk is important given the long-term health implications and the effectiveness of early intervention to prevent progression to cardiovascular disease.

## Introduction

Cardiovascular disease (CVD) is the leading cause of death globally. Over three-quarters of CVD deaths occur in low- and middle- income countries.^1^ In Sub-Saharan Africa, cardiovascular disease is the second leading cause of death, second only to human immunodeficiency virus (HIV).^2^ Risk factors include hypertension, smoking, diabetes mellitus, obesity, physical inactivity, and poor dietary patterns,^3^ often compounded by underlying risks such as poverty, stress, and hereditary factors.^1^

Although CVD and atherosclerosis clinically present in middle and late adulthood, atherosclerosis often starts in childhood, tracking to adulthood.^4,5^ Atherosclerosis can be prevented through dietary and physical activity interventions—potentially delaying or preventing CVD later in life.^4^

Pulse wave velocity (PWV), specifically carotid-femoral PWV is currently considered the non-invasive gold standard method of measuring arterial stiffness,^6^ and is widely accepted for the assessment of vascular stiffness in children and young adults by the American Heart Association.^7^ PWV is highly associated with the degree of atherosclerosis, as reduced elasticity leads to faster arterial pulse wave propagation. PWV is a potent predictor of incident cardiovascular events.^8–10^ Numerous non-invasive commercial devices are used to determine PWV, including the Vicorder, SphygmoCor, Complior, Arteriograph, and Mobil-O-Graph. These devices use different techniques for measuring arterial pulse such as applanation tonometry (PulsePen, SphygmoCor), automated oscillometry (Vicorder, Arteriograph, Mobil-O-Graph), or piezoelectric mechanotransducers (Complior). Gold-standard non-invasive devices include the SphygmoCor and Complior,^11^ however these techniques are heavily reliant on operator’s experience, and can be challenging in children.^12^ Instead, cuff-based devices using oscillometry require less operator skills, are less intrusive and therefore often preferable for children.^12,13^

To facilitate using PWV in pediatric clinical practice, a number of studies have constructed aortic PWV reference curves.^14–21^ However, most are in developed countries, with only one study of African children living in Sub-Saharan Africa.^21^ CVD and arterial stiffness are well known to differ across populations,^22^ with higher arterial stiffness in black adults.^23,24^

Our study defines reference values for PWV using the Vicorder device for a cohort of children and adolescents living in Cape Town, South Africa. We chose the Vicorder device to measure PWV given: (1) our low income setting; (2) the longitudinal nature of our study requiring high repeatability across multiple operators; (3) its use in several comparable PWV reference curve studies^14,16^; and (4) its producing comparable results in children to applanation tonometry, after path length correction.^13,25^ We used an anatomical PWV distance calculation that considers the growing child,^26^ using the widely accepted Lambda Mu and Sigma method (LMS) method.^27^ Our percentile curves will enable the calculation of gender-specific PWV z-scores for age and height in sub-Saharan African children. This tool will help to identify children at high risk of CVD, allowing early intervention to prevent premature CVD in adulthood.

Our cohort are from an area with high background rates of obesity, CVD and diabetes.^22^ We included both HIV unexposed and HIV exposed but uninfected children, given that sub-Saharan Africa has a high HIV-prevalence with an estimated 13.2 million HIV exposed but uninfected children,^28^ and an overall HIV prevalence of >20% in Eswatini (32%), Botswana (27%), and South Africa (22%).^28^

## Methods

### Setting and Design

We followed children at the Family Centre for Research with Ubuntu (FAMCRU) (Stellenbosch University and Tygerberg Children’s Hospital) from March 2014 to March 2020. Children were initially recruited as controls in longitudinal studies evaluating the rates of early atherosclerotic vascular disease in children living with perinatally-acquired HIV who initiated antiretroviral therapy (ART) in early infancy (National Institutes of Health grant number: 1R01HD083042) (Supplemental Figure 1). This control group—used in the current analysis—comprised 324 children with 959 longitudinal measurements.

Children originated from an urban region with high HIV prevalence. Given the potential for HIV to affect PWV,^29^ children living with HIV were excluded. Whether in utero exposure to HIV influences PWV is not well known, however, previous studies in this population (age 6–16 years) show no PWV difference between HIV-exposed and HIV-unexposed children.^30^ The inclusion of HIV exposed but uninfected children, together with HIV-unexposed children enhanced the generalizability of our results to sub-Saharan Africa.

Children comprised two ethnicity categories—“Black” (72%) and “Cape Mixed” (28%). The Cape Mixed ancestry group is a South African population group with Khoi, San, Malay, European, and African ancestry, unique to the Western Cape.^31,32^ Children had no known acute or chronic disease (such as high blood pressure, diabetes, obesity, chronic kidney disease, cardiac valve malformations), any chronic medications, or acute intercurrent infection.

The study was approved by Stellenbosch University’s Health Research Ethics Committee (Federal Wide Assurance Number: 00001372; Institutional Review Board Number: IRB0005239; Project approval number N12/11/076). Ongoing approval included annual data and participant safety review.

### Pulse Wave Velocity (PWV) Measurements

PWV was measured using the oscillometric Vicorder system (SMT Medical GmbH, Wurzberg, Germany),^33^ an automated and operator-independent device, allowing far greater reliability and repeatability than its counterparts for measuring PWV. A change in PWV indicates a change in aortic elasticity, a sensitive functional indicator of subclinical atherosclerosis.^34^ The function of the aorta is to receive the punch of pressure that exits the heart; to accommodate some of the pressure by stretching; and then to return that pressure slowly to the column of blood, thus converting quick turbulent flow into healthy (slower) laminar flow. When elasticity is lost, the aorta cannot efficiently absorb the impulse energy, with excess energy being retained as velocity. Thus, PWV varies according to the stiffness of the arterial wall: the stiffer the arterial wall, the greater the pulse wave propagation velocity.

PWV measurements were performed following the manufacturer instructions as previously described.^30^ With children lying supine, one cuff was placed on the neck over the right common carotid artery and the other around the upper right thigh over the femoral artery. The cuffs were then inflated to the set value, and carotid and femoral pulse waves were recorded in cycles of 3.5 s. The time delay between arrival of the pulse wave at the external carotid artery and then the femoral artery was recorded. This transit time represents the time taken to travel a fixed length of the aorta and proximal femoral artery. The velocity of the pulse wave is calculated as distance (*d*) divided by transit time (*t*) (PWV = *d*/*t*). To accommodate the small inherent variation in transit time from respiration and movement, each child had at least 100 measurements per visit, with the median transit time (*t*) used to calculate PWV. During the measurement process, children typically fell asleep.

#### Estimating the Distance Traveled by the Pulse Wave

The distance traveled by the pulse wave was estimated by surface anatomy measurements using a non-stretchable tape measure. In growing children, the method of estimating distance must be very precise, as aorta length changes with growth. Currently there is no consensus on how to accurately determine the real path of the arterial tree in children. To date, only one anatomical study provided guidance on which surface anatomy measurements most accurately represent the distance traveled by the intra-luminal pulse wave.^26^ Witbooi et al. measured true intra-arterial path lengths using multi-planar reformation imaging software for 3-dimensional reconstructions of 483 children scanned at different ages, comparing each with their own surface anatomy measurements using the same 3D reconstruction. They determined that the most accurate determination of distance traveled by the pulse wave is as follows: (1) measuring the distance (in mm) from the suprasternal notch (SSN) to the umbilicus, (2) continuing through the mid-inguinal crease to the femoral PWV recording site, (3) then subtracting an adjusted surface anatomy distance between the suprasternal notch and the angle of the mandible (PWV recording site in the neck), adjusted using the formula *y* = 4.791 + (1.0534*x). This translated into the following overall formula: (all measurements in cm):

$$\begin{array}{l} d\;=\;\left(\mathrm{SSN}-\;\mathrm{umb}\right)\;+\;\left(\mathrm{umb-il}\right)\;+\;\left(\mathrm{il-fem}\right)\;\\ \;\;\;\;\;\;-\left\{\left[\left(\mathrm{SSN-car}\right)*10*1.0534\right)+4.791]/10\right\} \end{array}$$

where *d*: distance traveled by pulse wave; SSN: suprasternal notch; umb: umbilicus; il: inguinal ligament; fem: femoral cuff; car: carotid cuff.

We used this validated distance estimation method for our analysis. In addition, we conducted sensitivity analyses for previously used formulas to estimate distance traveled to aid comparison with other PWV reference curves, specifically: (a) {(SSN-umb) + (umb-fem) − (SSN-car)}^14^; (b) {(car-fem)*0.8}^16,17^; (c) (car-fem)^21^; and (d) {(SSN to fem) − (SSN to car)}.^15^ These can be broadly grouped into two groups: anatomical methods, as in calculation (a) above, and straight-line methods, as in calculations (b–d) above. The anatomical method more accurately estimates the distance traveled by the pulse wave in children, by considering the changing height and greater variation in body segments in proportions with age in children.^13^ The straight-line method, in comparison, measures the distance between the two cuffs and some apply a correction factor of 0.8. This method is common practice in non-growing adults in whom the pulse wave distance remains constant over time. This latter method is not appropriate in children, where the distance traveled by the pulse wave changes with growth.

### Inclusion and Exclusion Criteria

All children with pulse wave velocity measurements over the 6-year follow-up period were eligible for inclusion. Information on cardiometabolic indicators was collected at routine annual clinic appointments. However, children had varying numbers of datapoints and timepoints due to resource limitations, children lost to follow up, and missed clinic appointments.

### Statistical Analysis

Descriptive analysis includes mean anthropometric and cardiovascular (PWV, heart rate, mean arterial pressure, systolic and diastolic BP) measures divided into three age groups—6–9, 9–12, and 12–16 years, to understand change over time. We compared PWV by gender, ethnicity and HIV exposure status over time using linear mixed models with random intercepts, to understand potential differences within the cohort.

Centile curves were constructed using the Lamda-Mu-Sigma (LMS) method and GAMLSS (Generalized Additive models for Location Scale and Shape).^35^ The LMS method^27^ is recommended by the World Health Organization and accounts for nonlinearity and skewed distributions. The LMS method constructs centile curves by summarizing the distribution according to three parameters: *L*—the power of the Box-Cox transformation (a measure of skewness); *M*—the median of PWV within the age (or height) band, and *S*—the coefficient of variation within the band. The LMS method has been used to calculate PWV reference curves in children from other populations,^14–18,20^ allowing for suitable comparisons of centile curves. In GAMLSS, the LMS method is renamed the Box-Cox Cole and Green (BCCG) distribution.^36^ We also considered extensions of the LMS method that adjust for kurtosis—notably Box-Cox t (BCT), that adjusts for kurtosis based on the *t* distribution, and the Box-Cox Power Exponential (BCPE), that adjusts for kurtosis based on the power exponential distribution. For each gender and age/height, we used the optimal model based on the lowest generalized AIC (GAIC),^37^ and providing tau—the kurtosis parameter—was positive. Cubic spline (P-spline) smoothing was used to estimate the curve for each parameter as a smooth function of height or age. The BCT distribution was the best fit for males by height and age, while the BCCG distribution was the best fit for females by height and age.

*Z*-scores for an individual child can be calculated according to the following formulas:

z=(PWVmeasureM)L-1LSforL≠0andz=log(PWVmeasureM)SforL=0.

In the case of a BCT model, the random variable *Z* is assumed to follow a *t* distribution with degrees of freedom Tau > 0, treated as a continuous parameter.

Statistical analysis was performed in RStudio^38^ using the GAMLSS^35^ package. GAMLSS is theoretically able to model longitudinal data, however mixed effects models incorporating child as a random effect produced irregular centiles, thus we employed cross-sectional GAMLSS using all measurements in the dataset. In practice, longitudinal data are often used to construct size charts, by ignoring the repeated measures in the data.^39^ The centiles will be unbiased provided the frequency of measurements is independent of previous measurements, as is the case here.^40,41^ Residual diagnostics (worm plots and *Q* statistics) were used to check for appropriateness of model fit.

We ran sensitivity analyses adjusting the calculation of PWV distance, using a similar GAMLSS approach, to aid comparisons with other PWV centile curves.

## Results

### Characteristics of Children

Three hundred and twenty four children (48% female, 52% male) were included in the analysis. 72% were Black (*n* = 233), and 28% Mixed race (*n* = 91). 51% of children were HIV unexposed (*n* = 164) and 49% HIV exposed uninfected (*n* = 160). Supplemental Table 1 outlines cross-sectional characteristics of the cohort by gender.

Participants were between 6.3 and 15.6 years of age. There were 959 total PWV measurements over the study period (457 female and 502 male), with a median of 3 measurements per participant. The median (IQR) follow-up time was 2.1 (IQR 1.0, 3.0) years. Table 1 outlines the longitudinal characteristics of the cohort, split by gender and three age groups. Median PWV did not change significantly between the ages of 6–9 and 12–16 regardless of gender. Linear mixed models showed no significant difference in mean PWV by gender (*P* = .477), ethnicity (*P* = .596) and HIV exposure status (*P* = .162), after adjusting for age. Supplemental Figure 2 shows the unadjusted progression of PWV by age, split by gender, ethnicity, and HIV exposure status.

**Table 1.** Longitudinal Characteristics of the Cohort by Gender, Divided into Three Age Groups. Median (IQR) are Reported.

Characteristic:	Age group (years)		
	6–8.99 years	9–11.99 years	12–16 years
Females			
Number of measurements	111	231	115
Number of children	80	125	66
Anthropometrics:			
Age (yr)—median (IQR)	8.3 (7.7, 8.7)	10.5 (9.8, 11.1)	13.4 (12.7, 14.0)
Height (cm)—median (IQR)	124.9 (120.9, 130.3)	138.4 (133.1, 144.5)	152.7 (149.1, 157.9)
Weight (kg)—median (IQR)	25.4 (22.5, 28.0)	32.8 (28.3, 39.5)	48.0 (41.8, 56.3)
Tanner stage—median (IQR)	1.0 (1.0, 1.0)	1.0 (1.0, 2.0)	3.0 (2.0, 3.0)
Cardiovascular measures:			
PWV—median (IQR)	5.01 (4.63, 5.34)	4.80 (4.46, 5.27)	5.00 (4.71, 5.30)
Heart rate—median (IQR)	89.0 (81.0, 99.0)	81.0 (75.0, 87.5)	76.0 (71.0, 82.0)
Mean arterial pressure—median (IQR)	75.7 (70.0, 80.2)	80.7 (75.8, 85.4)	87.2 (83.8, 90.6)
Systolic BP—median (IQR)	96.0 (91.8, 100.0)	100.0 (97.3, 107.3)	109.3 (105.8, 114.0)
Diastolic BP—median (IQR)	65.0 (58.5, 70.0)	70.0 (62.7, 78.0)	76.7 (72.0, 80.0)
Males			
Number of measurements	115	268	119
Number of children	91	141	70
Anthropometrics:			
Age (yr)—median (IQR)	8.4 (7.7, 8.6)	10.6 (9.8, 11.1)	13.2 (12.6, 13.7)
Height (cm)—median (IQR)	125.4 (121.5, 130.0)	135.9 (131.3, 141.5)	150.5 (143.2, 157.3)
Males			
Weight (kg)—median (IQR)	24.9 (22.2, 28.3)	30.4 (27.3, 35.1)	41.6 (34.9, 48.2)
Tanner stage—median (IQR)	1.0 (1.0, 1.0)	1.0 (1.0, 2.0)	3.0 (2.0, 3.0)
Cardiovascular measures:			
PWV—median (IQR)	4.88 (4.49, 5.26)	4.83 (4.52, 5.20)	4.86 (4.53, 5.24)
Heart rate—median (IQR)	82.0 (75.0, 91.0)	77.0 (70.0, 85.0)	69.0 (63.5, 75.0)
Mean arterial pressure—median (IQR)	74.6 (69.8, 80.1)	79.6 (75.1, 84.2)	84.3 (79.7, 89.1)
Systolic BP—median (IQR)	98.0 (92.7, 102.7)	100.0 (96.0, 106.0)	108.0 (102.0, 111.3)
Diastolic BP—median (IQR)	62.0 (58.0, 70.0)	68.7 (63.7, 74.3)	74.0 (67.5, 78.7)

Anthropometric and cardiovascular measurements are simple medians, and do not take into account multiple measurements per child.

Abbreviation: PWV, pulse wave velocity.

**Figure 1.**
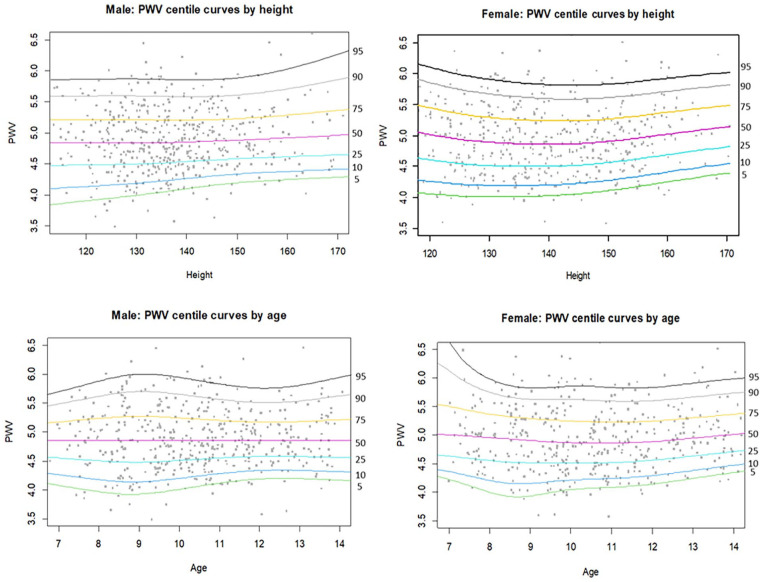
Percentiles of pulse wave velocity for males and females by height and age. The curves were derived using GALMSS (31) in RStudio (35). The optimal distribution for males by age and height was BCT, while the BCCG distribution was the best fit for females by age and height. Abbreviations: PWV, pulse wave velocity; GALMSS, generalized additive model for location, scale and shape; BCT, box-cox *t* distribution; BCCG, box-cox cole and green distribution.

Percentile curves from the GAMLSS analysis are in Figure 1. The predicted percentile curves are relatively flat in line with descriptive analysis, with a slight increase towards greater height and age. For males, median predicted PWV at 120 cm was 4.84 m/s, rising slightly to 4.96 m/s at 170 cm. Between 7 and 14 years of age, median predicted PWV in males remained flat at 4.86 m/s. In females, median predicted PWV at 120 cm was 5.02 m/s, rising slightly to 5.14 m/s at 170 cm. Likewise, there was no significant change in median predicted PWV in females from age 7 (PWV: 5.01 m/s) to age 14 (PWV: 5.01 m/s). Model fit statistics were within acceptable ranges. Supplemental Tables 2–5 provide parameter and centile tables by height and age for males and females.

Supplemental Figure 3 presents a sensitivity analysis adjusting the PWV distance estimation method. Using straight line methods—as in (car-fem)*0.8—results in an apparent increase in PWV in both males and females by height, with a median predicted PWV at 120 cm of 4.47 m/s rising to 5.04 m/s by 170 cm in males (4.63 m/s to 4.99 m/s in females). This apparent increase is also seen by age, with an increase in PWV from age 11 onwards. This figure demonstrates the significant effect that the PWV distance estimation method has on PWV centile curves. Using a straight line distance estimation method results in a misleading distortion of PWV centile curves for growing children.

## Discussion

### Statement of Principal Findings

This study provides gender, age, and height specific anatomically-based PWV reference curves using the Vicorder device^33^ based on the largest cohort of African children published to date. Given the importance of identifying children at high risk of CVD, this study provides a valuable contribution to the literature, highlighting the importance of population-specific reference curves and enabling the calculation of appropriate *Z*-scores of PWV in African children. Our results indicate that African children have a relatively flat PWV between the ages of 7 and 14 years old, and between 120 and 170 cm standing height. Sensitivity analysis shows the significant effect the PWV distance estimation method has on PWV centile curves, with straight line estimation methods distorting predicted percentile curves.

### Comparison with Other Studies

Table 2 summarizes existing PWV reference studies in children. A comparison of PWV curves should be interpreted cautiously and will be discussed based on the following considerations: (1) Differences in the PWV distance estimation method; (2) Differences in ethnicity and population; (3) Differences in the recording device; (4) Differences in the statistical method to derive the reference curves. These factors may explain some of the variability seen across studies.

**Table 2.** Summary of Pediatric Aortic PWV Studies Reference Studies (m/s).

First author	Year	Country	Study design	Sample size (*n*)	Age range (years)	Device	PWV calculation	Statistical method
Thurn et al. (14)	2015	Germany/Turkey	Cross-sectional	1003	6–18	Vicorder, oscillometry	PWV = [(SSN to umb) + (umb to fem) − (SSN to car)]/Transit time	LMS method
Fischer et al. (16)	2012	Germany	Cross-sectional	314	8–17	Vicorder, oscillometry	PWV = [(car to fem)*0.8] /Transit time	LMS method—age and height
Mora-Urda et al. (17)	2017	Spain	Cross-sectional	350	8–11	SphygmoCor, tonometry	PWV = [(car to fem)*0.8] /Transit time	LMS method—age and height
Silva et al. (21)	2016	Angola	Cross-sectional	157	8–10.5	Complior, Tonometry	PWV = (car to fem)/Transit time	Quantile regression—age and height
Reusz et al. (15)	2010	Hungary	Cross-sectional	1008	7–19	Pulsepen, Tonometry	PWV = [(SSN to fem) − (SSN to car)]/Transit time	LMS method—age and height
Hidvégi et al. (20)	2012	Hungary	Cross-sectional	3374	3–18	Arteriograph, oscillometry	PWV = SSN–pubic bone/transit time	LMS method—age
Elmenhorst et al. (18)	2015	Germany	Cross-sectional	2227	8–22	Mobil-O-Graph, oscillometry	Inbuilt ARCSolver algorithm based on 24 h blood pressure values	LMS method—age and height

Abbreviations: PWV, pulse wave velocity; SSN, suprasternal notch; umb, umbilicus; il, inguinal ligament; fem, femoral cuff recording site; car, carotid cuff recording site.

#### Methods Used to Estimate Distance Traveled by the Pulse Wave in Growing Children

As explained earlier, PWV is a velocity calculated using the formula: distance traveled/transit time. In adults, who are not growing, anatomical distance traveled by the pulse wave is assumed to be static; therefore, any change in transit time indicates a change in vascular elasticity. However, in growing children, anatomical distance traveled by the pulse wave changes over time and therefore the method to estimate distance must be anatomically accurate. For our reference curves, we used the anatomical method. Our sensitivity analysis (Supplemental Figure 3) highlights the significant distortion of PWV centile curves when using straight line distance estimation methods, which do not follow the anatomical pathway in growing children. Had we employed a straight line distance estimation method, our PWV curves would artefactually and spuriously appear to increase with height and age.

**Figure 2.**
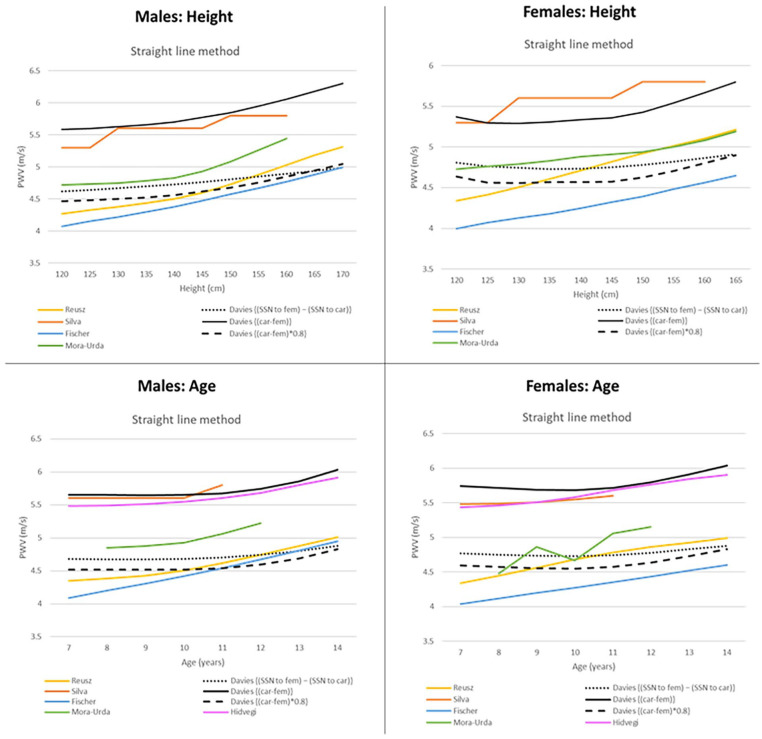
Comparison of published 50th percentile reference curves that use straight line PWV distance estimation methods. Straight line methods measure the distance between the two cuffs and some apply a correction factor of 0.8. Our sensitivity analysis using straight line PWV distance estimation methods is included for comparison purposes. The following distance estimation methods have been used in each published paper: • {(SSN to fem) − (SSN to car)}: Used by Reusz et al.^15^ • {(car-fem)}: Used by Silva et al.^21^ • {(car-fem)*0.8}: Used by Fischer et al. and Mora-Urda et al.^16,17^ • {SSN–pubic bone}: Used by Hidvegi et al.^20^ Elmenhorst et al.^18^ has been excluded as they use the ARCSolver algorithm from the Mobil-O-Graph. Hidvegi et al.^20^ only constructed curves by age; thus no height curve is available. Abbreviations: PWV, pulse wave velocity; SSN, suprasternal notch; umb, umbilicus; fem, femoral cuff; car, carotid cuff.

Several existing PWV reference studies do not use the anatomical method, and instead use straight-line methods.^15–17,20,21^ Figure 2 provides a comparison of our 50th percentile sensitivity curves (using straight line distance estimation methods) with the 50th percentile curves of published studies that use straight line PWV distance estimation methods. Our sensitivity curves are in line with other studies reporting an apparent increase in PWV with height. Likewise, our sensitivity curves align with other studies reporting a relatively flat PWV up to age 10/11. A recent study by Reusz et al.^42^ re-evaluated their 2010 PWV percentile norms^15^ using a different PWV calculation of (car-fem)*0.8, and in a subset of children (*n* = 31) compared the distances to Magnetic Resonance Imaging (MRI)-recorded intra-arterial distance. They found that (car-fem)*0.8 overestimates the true path length in children >14 years old compared with {(SSN to fem) − (SSN to car)}, with marginal differences in younger children. However, both measures exclude the distance via the umbilicus. The MRI sub-study showed good agreement between {(SSN to fem) − (SSN to car)} and MRI analysis, however distances were only validated in 31 MRIs with a mean age of 12 years (standard deviation 4.4 years), and in children with medically justified MRIs. In contrast, Witbooi et al. used 483 computed tomography (CT) scans.^26^ In addition, the studies were conducted in different populations.

#### Different Norms in Different Ethnic Groups

**Figure 3.**
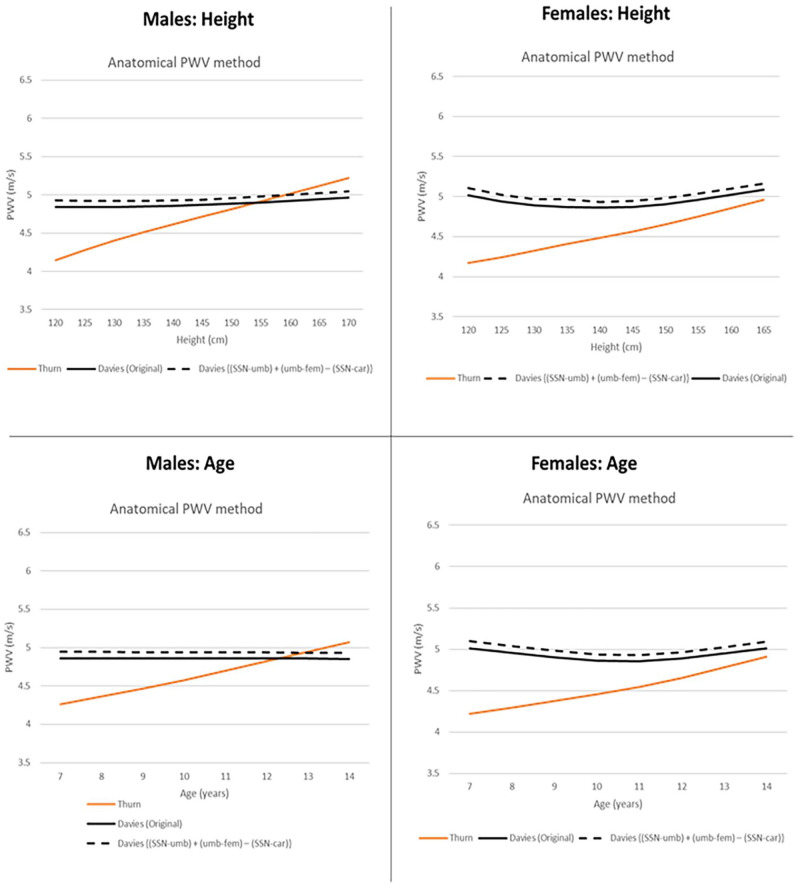
50th percentile curves of African children (our data) compared to Caucasian children, using the anatomical PWV distance estimation method. The anatomical method estimates the distance traveled by the pulse wave in children by considering the changing height and greater variation in body segments in proportions with age.^13^ {(SSN-umb) + (umb-fem) − (SSN-car)}: Used by Thurn et al.^14^ Davies (Original formula): *d* = (SSN-umb) + (umb-il) + (il-fem) − {[(SSN-car)*10*1.0534) + 4.791]/10} Abbreviations: PWV, pulse wave velocity; d, distance traveled by pulse wave; SSN, suprasternal notch; umb, umbilicus; il, inguinal ligament; fem, femoral cuff; car, carotid cuff.

Remarkably, our study demonstrates that African children have a relatively flat PWV throughout childhood and early adolescence. Thurn et al.^14^ used an appropriate distance estimation method that follows the anatomical pathway of the pulse wave in Caucasian children. Figure 3 compares the 50th percentile curves of African children (our data) and Caucasian children (Thurn et al.).^14^ The differences seen can be attributed to differences in ethnicity and population, as the same Vicorder device and LMS calculation were used in both populations. Race is a well-known variable influencing PWV, and male African children have increased arterial stiffness in all sections of the arterial tree compared with Caucasian counterparts.^43^ This is important given that the prevalence of hypertension is highest in Africa and an emerging public health burden in sub-Saharan Africa.^44^ Only one PWV reference study was conducted in African children (Silva et al.).^21^ This study reports a stable PWV by age in prepubertal healthy black African schoolchildren in Angola until age 10, with only a slight increase at age 11. This is similar to our observations when we used their PWV distance estimation method (Figure 2). In addition, our study uses a larger sample size of 324 children versus 157 used by Silva et al.^21^

#### Different Recording Devices

Published reference curves also use different recording devices, although few have been validated in children. The Pulsepen, Sphygmocor and Vicorder device all produce comparable results in children, after path length correction.^25^ A further study comparing the Vicorder and Sphygmocor found that the best agreement between devices is obtained using a path length that most accurately describes the aortic tree.^13^

### Strengths and Limitations of the Study

Strengths of the study include accurate measurements of PWV, given that each child had at least 100 measurements during each PWV recording. In addition, we adjusted the calculation of PWV obtained from the Vicorder system to more accurately reflect the anatomical distance traveled by the pulse wave in children,^26^ thereby overcoming shortcomings of the Vicorder system.^45^ However, one cannot exclude the possibility of different results should another device be used to measure PWV.

Our sample size is small compared with other PWV reference studies in Caucasians. However, it is the largest known study in Africa. We include a mixed group of children of HIV exposed and HIV unexposed children and young adolescents from Black and Mixed ethnicity. Linear mixed models showed that the mean PWV did not differ across these groups over time, giving confidence in the validity of our approach.

The estimation of percentile curves did not account for the longitudinal nature of the dataset. However, given that the value of measurements did not affect the frequency of future measurements, bias in the centiles is negligible.^36,40^ There is greater imprecision in our dataset at the extremes of the height and age ranges based on reduced measurements. To counteract this limitation, we truncated the resulting curves to improve precision of the centile estimates at the extremes.^46^

### Generalizability

Our findings are generalizable for measuring PWV in sub-Saharan African children using the Vicorder device and the anatomical pathway to account for growth. Our data may not extrapolate to African immigrants living in developed countries, whose environment and exposures are very different.

Our dataset contained children with study visits ranging between 6 and 16 years old and from Tanner Stage 1 to Tanner Stage 5. However, as the median age at last visit was 12.4 (IQR 10.8, 13.5) years, at the last PWV measurement only 9% had reached Tanner Stages 4 or 5. Therefore, our findings are applicable to the pre-pubertal and early pubertal period. Further research is necessary to evaluate PWV at the end of puberty in this population.

## Conclusion

This study defines reference values for PWV for African children, based on a cohort of children and adolescents living in Cape Town, South Africa, using the oscillometric Vicorder device. These gender-specific percentiles for age and height will allow accurate surveillance of arterial elasticity in African children over time. The identification of children at high risk is important given the long-term health implications and the effectiveness of early intervention to prevent progression to CVD. Furthermore, this study highlights the need for population-specific PWV reference curves to guide health policy.

## Supplemental Material

sj-docx-1-ang-10.1177_00033197251314218 – Supplemental material for Carotid-Femoral Pulse Wave Velocity in Children in South Africa: Reference Values for the Vicorder Device Supplemental material, sj-docx-1-ang-10.1177_00033197251314218 for Carotid-Femoral Pulse Wave Velocity in Children in South Africa: Reference Values for the Vicorder Device by Claire Davies, Florin Vaida, Kennedy Otwombe, Mark F. Cotton, Sara Browne and Steve Innes in Angiology
