# Supplementary material for: Carotid-Femoral Pulse Wave Velocity in Children in South Africa: Reference Values for the Vicorder Device
Source: Angiology. 2025 Jan 23;77(2):229–40. doi: 10.1177/00033197251314218 (PMC12779766; doi:10.1177/00033197251314218)
Supplement: sj-docx-1-ang-10.1177_00033197251314218 – Supplemental material for Carotid-Femoral Pulse Wave Velocity in Children in South Africa: Reference Values for the Vicorder Device [file sj-docx-1-ang-10.1177_00033197251314218.docx]

**Supplementary Figure 1: Participants recruited for PWV measurement**

HIV Unexposed

HIV Exposed Uninfected

Additional participants recruited: n = 94

6-8 years old: n = 16

8-10 years old: n = 40

10-12 years old: n = 29

12-16 years old: n = 9

Completed parent trial: n = 56

CHER: n = 23

1104: n = 33

Completed parent trial: n = 70

CHER: n = 32

1104: n = 38

Additional participants recruited: n = 104

6-8 years old: n = 27

8-10 years old: n = 38

10-12 years old: n = 25

12-16 years old: n = 14

Performed PWV measurements:

n = 164

Performed PWV measurements:

n = 160

PWV: Pulse wave velocity; HIV: Human immunodeficiency virus; CHER: Children with HIV Early Antiretroviral Therapy (CHER) trial

**Supplementary Table 1: Cross-sectional characteristics of the cohort split by gender, by total number of children**

|  | **Total**  **(n = 324)** | **Female**  **(n = 170)** | **Male**  **(n = 154)** |
| --- | --- | --- | --- |
|  | **(n, %)** | **(n, %)** | **(n, %)** |
| HIV status |  |  |  |
| HIV unexposed | 164 (51) | 72 (47) | 92 (54) |
| HIV exposed uninfected | 160 (49) | 82 (53) | 78 (46) |
| Ethnicity |  |  |  |
| Black | 233 (72) | 109 (71) | 124 (73) |
| Mixed race | 91 (28) | 45 (29) | 46 (27) |
| Family history of diabetes |  |  |  |
| Yes | 77 (24) | 40 (26) | 37 (22) |
| No | 208 (64) | 98 (64) | 110 (65) |
| Missing | 39 (12) | 16 (10) | 23 (14) |
| Family history of cardiovascular disease |  |  |  |
| Yes | 48 (15) | 20 (13) | 28 (16) |
| No | 235 (73) | 118 (77) | 117 (69) |
| Missing | 41 (13) | 16 (10) | 25 (15) |

HIV: Human immunodeficiency virus

**Supplementary Figure 2: Unadjusted means (with 95% CI) of pulse wave velocity (PWV) by age split by gender, ethnicity and HIV group. Means are calculated for each yearly age band.**

PWV: Pulse wave velocity

HIV unexposed uninfected

HIV exposed uninfected

HIV: Human immunodeficiency virus

**Supplementary Figure 3: Sensitivity Analysis of predicted 50^th^ PWV percentile curves resulting from anatomical vs straight line methods of estimating distance travelled by the pulse wave in growing children**


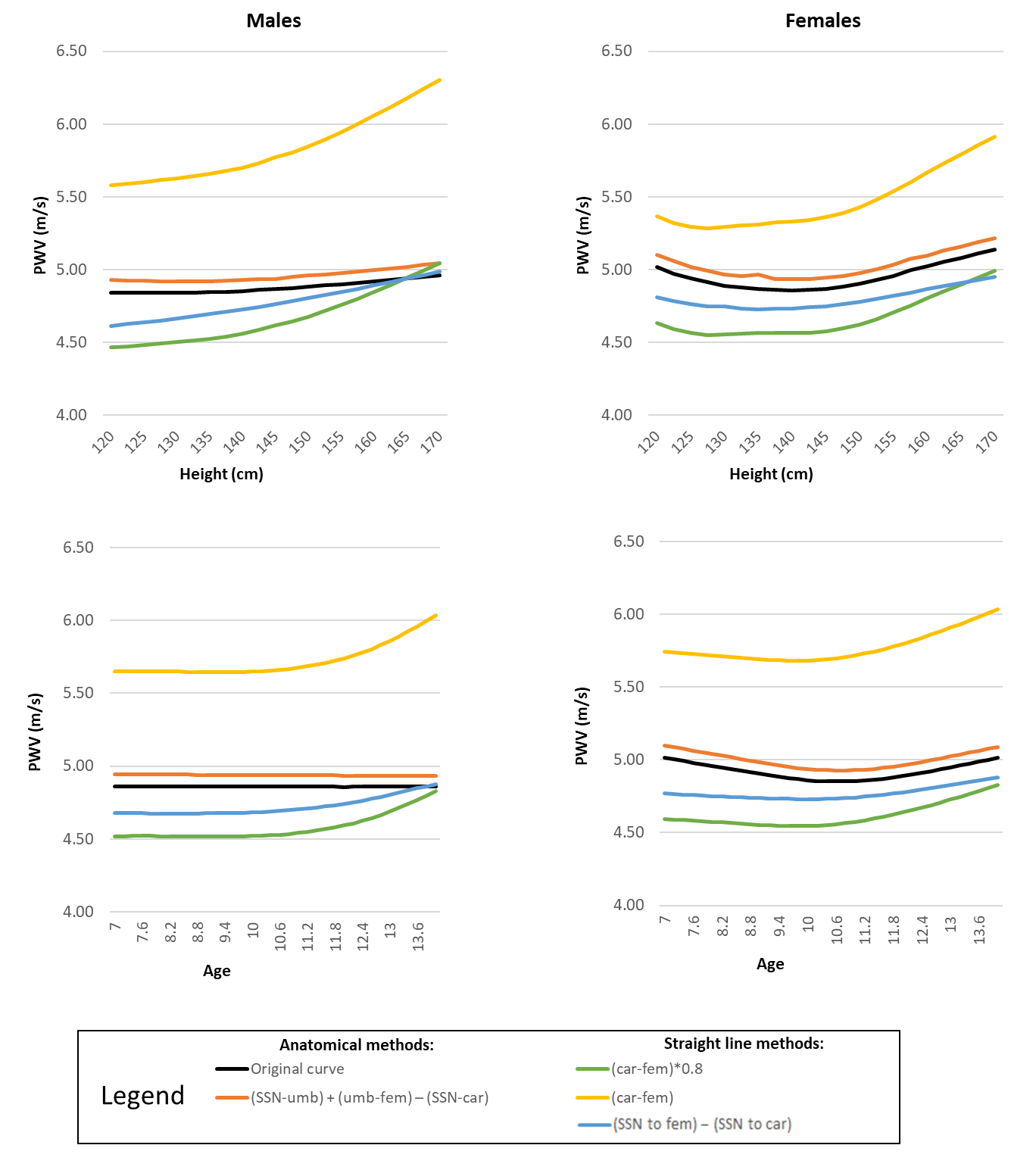


PWV median curves are all produced using the GALMSS package (31) in RStudio (35).

PWV: Pulse wave velocity; GALMSS: generalized additive model for location, scale and shape; SSN: suprasternal notch; umb: umbilicus; fem: femoral cuff; car: carotid cuff

**Supplementary Table 2: Box Cox t parameters and percentiles for males by height**

|  | **Parameters** | | | | **Centiles of PWV (m/s)** | | | | | | |
| --- | --- | --- | --- | --- | --- | --- | --- | --- | --- | --- | --- |
| **Height (cm)** | **L (Lambda)** | **M (mu)** | **S (sigma)** | **T (Tau)** | **5** | **10** | **25** | **50** | **75** | **90** | **95** |
| 120 | 0.573 | 4.842 | 0.106 | 6.795 | 3.91 | 4.14 | 4.48 | 4.84 | 5.21 | 5.60 | 5.86 |
| 125 | 0.286 | 4.841 | 0.106 | 7.641 | 3.95 | 4.16 | 4.49 | 4.84 | 5.21 | 5.60 | 5.87 |
| 130 | -0.006 | 4.841 | 0.104 | 8.612 | 3.99 | 4.19 | 4.50 | 4.84 | 5.21 | 5.60 | 5.87 |
| 135 | -0.304 | 4.845 | 0.102 | 9.729 | 4.05 | 4.22 | 4.51 | 4.84 | 5.21 | 5.59 | 5.86 |
| 140 | -0.608 | 4.853 | 0.098 | 11.016 | 4.10 | 4.27 | 4.54 | 4.85 | 5.21 | 5.58 | 5.85 |
| 145 | -0.916 | 4.868 | 0.096 | 12.503 | 4.16 | 4.31 | 4.56 | 4.87 | 5.21 | 5.59 | 5.86 |
| 150 | -1.231 | 4.883 | 0.095 | 14.219 | 4.20 | 4.34 | 4.58 | 4.88 | 5.23 | 5.61 | 5.89 |
| 155 | -1.550 | 4.901 | 0.096 | 16.207 | 4.23 | 4.36 | 4.60 | 4.90 | 5.25 | 5.65 | 5.94 |
| 160 | -1.875 | 4.920 | 0.098 | 18.512 | 4.25 | 4.38 | 4.62 | 4.92 | 5.29 | 5.71 | 6.03 |
| 165 | -2.205 | 4.940 | 0.100 | 21.190 | 4.27 | 4.40 | 4.63 | 4.94 | 5.32 | 5.78 | 6.14 |
| 170 | -2.540 | 4.962 | 0.103 | 24.305 | 4.29 | 4.42 | 4.65 | 4.96 | 5.36 | 5.86 | 6.26 |

Calculation of z-scores is according to the formula: $z=\frac{(\frac{PWV measure}{M})^{L}-1}{LS} \mathrm{for}L\neq0$ and $z=\frac{\log\left( \frac{PWV measure}{M} \right)}{S} for L=0$, with the random variable Z assumed to follow a *t* distribution with degrees of freedom Tau, treated as a continuous parameter.

PWV: Pulse wave velocity

**Supplementary Table 3: Box Cox t parameters and percentiles for males by age**

|  | **Parameters** | | | | **Centiles of PWV (m/s)** | | | | | | |
| --- | --- | --- | --- | --- | --- | --- | --- | --- | --- | --- | --- |
| **Age** | **L (Lambda)** | **M (mu)** | **S (sigma)** | **T (Tau)** | **5** | **10** | **25** | **50** | **75** | **90** | **95** |
| 7.0 | 0.571 | 4.861 | 0.091 | 9.073 | 4.08 | 4.27 | 4.55 | 4.86 | 5.18 | 5.49 | 5.70 |
| 7.5 | 0.444 | 4.861 | 0.099 | 9.094 | 4.02 | 4.22 | 4.53 | 4.86 | 5.21 | 5.55 | 5.79 |
| 8.0 | 0.313 | 4.860 | 0.107 | 9.117 | 3.97 | 4.18 | 4.50 | 4.86 | 5.24 | 5.62 | 5.88 |
| 8.5 | 0.177 | 4.860 | 0.114 | 9.140 | 3.93 | 4.15 | 4.49 | 4.86 | 5.26 | 5.67 | 5.96 |
| 9.0 | 0.038 | 4.860 | 0.116 | 9.164 | 3.93 | 4.14 | 4.48 | 4.86 | 5.27 | 5.70 | 6.00 |
| 9.5 | -0.106 | 4.860 | 0.113 | 9.189 | 3.96 | 4.16 | 4.49 | 4.86 | 5.26 | 5.69 | 5.99 |
| 10.0 | -0.253 | 4.859 | 0.107 | 9.214 | 4.01 | 4.20 | 4.51 | 4.86 | 5.24 | 5.65 | 5.94 |
| 10.5 | -0.404 | 4.859 | 0.101 | 9.240 | 4.07 | 4.24 | 4.53 | 4.86 | 5.22 | 5.61 | 5.89 |
| 11.0 | -0.559 | 4.859 | 0.095 | 9.267 | 4.12 | 4.28 | 4.55 | 4.86 | 5.20 | 5.57 | 5.83 |
| 11.5 | -0.717 | 4.859 | 0.090 | 9.295 | 4.16 | 4.32 | 4.57 | 4.86 | 5.18 | 5.53 | 5.78 |
| 12.0 | -0.878 | 4.859 | 0.087 | 9.323 | 4.19 | 4.34 | 4.58 | 4.86 | 5.17 | 5.51 | 5.76 |
| 12.5 | -1.043 | 4.859 | 0.086 | 9.352 | 4.20 | 4.34 | 4.58 | 4.86 | 5.17 | 5.51 | 5.77 |
| 13.0 | -1.212 | 4.859 | 0.088 | 9.381 | 4.20 | 4.34 | 4.58 | 4.86 | 5.18 | 5.54 | 5.81 |
| 13.5 | -1.383 | 4.859 | 0.091 | 9.412 | 4.18 | 4.33 | 4.57 | 4.86 | 5.20 | 5.58 | 5.87 |
| 14.0 | -1.558 | 4.859 | 0.095 | 9.443 | 4.17 | 4.31 | 4.56 | 4.86 | 5.21 | 5.62 | 5.95 |

Calculation of z-scores is according to the formula: $z=\frac{(\frac{PWV measure}{M})^{L}-1}{LS} \mathrm{for}L\neq0$ and $z=\frac{\log\left( \frac{PWV measure}{M} \right)}{S} for L=0$, with the random variable Z assumed to follow a *t* distribution with degrees of freedom Tau, treated as a continuous parameter.

PWV: Pulse wave velocity

**Supplementary Table 4: LMS parameters and percentiles for females by height**

|  | **Parameters** | | | **Centiles of PWV (m/s)** | | | | | | |
| --- | --- | --- | --- | --- | --- | --- | --- | --- | --- | --- |
| **Height (cm)** | **L (Lambda)** | **M (mu)** | **S (sigma)** | **5** | **10** | **25** | **50** | **75** | **90** | **95** |
| 120 | 0.364 | 5.016 | 0.124 | 4.06 | 4.26 | 4.61 | 5.02 | 5.45 | 5.86 | 6.11 |
| 125 | 0.327 | 4.939 | 0.121 | 4.02 | 4.21 | 4.55 | 4.94 | 5.35 | 5.75 | 5.99 |
| 130 | 0.289 | 4.890 | 0.118 | 4.00 | 4.19 | 4.51 | 4.89 | 5.29 | 5.67 | 5.91 |
| 135 | 0.252 | 4.887 | 0.115 | 4.03 | 4.21 | 4.52 | 4.89 | 5.28 | 5.65 | 5.88 |
| 140 | 0.214 | 4.858 | 0.112 | 4.02 | 4.20 | 4.50 | 4.86 | 5.24 | 5.60 | 5.82 |
| 145 | 0.177 | 4.870 | 0.109 | 4.06 | 4.23 | 4.52 | 4.87 | 5.24 | 5.59 | 5.81 |
| 150 | 0.139 | 4.904 | 0.107 | 4.11 | 4.27 | 4.56 | 4.90 | 5.27 | 5.61 | 5.83 |
| 155 | 0.102 | 4.958 | 0.104 | 4.17 | 4.34 | 4.62 | 4.96 | 5.32 | 5.66 | 5.87 |
| 160 | 0.064 | 5.023 | 0.101 | 4.25 | 4.41 | 4.69 | 5.02 | 5.38 | 5.72 | 5.93 |
| 165 | 0.027 | 5.083 | 0.099 | 4.32 | 4.48 | 4.76 | 5.08 | 5.43 | 5.77 | 5.98 |
| 170 | -0.010 | 5.139 | 0.096 | 4.39 | 4.54 | 4.82 | 5.14 | 5.48 | 5.81 | 6.02 |

Calculation of z-scores is according to the formula: $z=\frac{(\frac{PWV measure}{M})^{L}-1}{LS} \mathrm{for}L\neq0$ and $z=\frac{\log\left( \frac{PWV measure}{M} \right)}{S} for L=0$

PWV: Pulse wave velocity

**Supplementary Table 5: LMS parameters and percentiles for females by age**

|  | **Parameters** | | | **Centiles of PWV (m/s)** | | | | | | |
| --- | --- | --- | --- | --- | --- | --- | --- | --- | --- | --- |
| **Age** | **L (Lambda)** | **M (mu)** | **S (sigma)** | **5** | **10** | **25** | **50** | **75** | **90** | **95** |
| 7.0 | -2.600 | 5.015 | 0.128 | 4.24 | 4.37 | 4.64 | 5.01 | 5.53 | 6.20 | 6.78 |
| 7.5 | -0.720 | 4.986 | 0.125 | 4.12 | 4.29 | 4.59 | 4.99 | 5.44 | 5.91 | 6.23 |
| 8.0 | 0.735 | 4.958 | 0.122 | 3.99 | 4.20 | 4.56 | 4.96 | 5.37 | 5.75 | 5.98 |
| 8.5 | 1.440 | 4.931 | 0.119 | 3.92 | 4.15 | 4.53 | 4.93 | 5.32 | 5.66 | 5.86 |
| 9.0 | 1.266 | 4.903 | 0.117 | 3.94 | 4.15 | 4.51 | 4.90 | 5.28 | 5.62 | 5.82 |
| 9.5 | 0.577 | 4.878 | 0.114 | 4.00 | 4.19 | 4.51 | 4.88 | 5.26 | 5.61 | 5.83 |
| 10.0 | -0.081 | 4.860 | 0.112 | 4.05 | 4.21 | 4.51 | 4.86 | 5.24 | 5.61 | 5.85 |
| 10.5 | -0.354 | 4.852 | 0.110 | 4.07 | 4.23 | 4.51 | 4.85 | 5.23 | 5.61 | 5.85 |
| 11.0 | -0.368 | 4.855 | 0.108 | 4.09 | 4.24 | 4.52 | 4.85 | 5.23 | 5.59 | 5.83 |
| 11.5 | -0.369 | 4.868 | 0.106 | 4.11 | 4.26 | 4.54 | 4.87 | 5.23 | 5.59 | 5.83 |
| 12.0 | -0.460 | 4.889 | 0.104 | 4.15 | 4.30 | 4.56 | 4.89 | 5.25 | 5.61 | 5.84 |
| 12.5 | -0.638 | 4.917 | 0.102 | 4.19 | 4.34 | 4.60 | 4.92 | 5.28 | 5.64 | 5.87 |
| 13.0 | -0.867 | 4.948 | 0.100 | 4.24 | 4.38 | 4.63 | 4.95 | 5.31 | 5.67 | 5.91 |
| 13.5 | -1.085 | 4.981 | 0.099 | 4.29 | 4.42 | 4.67 | 4.98 | 5.34 | 5.71 | 5.96 |
| 14.0 | -1.262 | 5.013 | 0.097 | 4.33 | 4.47 | 4.71 | 5.01 | 5.37 | 5.74 | 5.99 |

Calculation of z-scores is according to the formula: $z=\frac{(\frac{PWV measure}{M})^{L}-1}{LS} \mathrm{for}L\neq0$ and $z=\frac{\log\left( \frac{PWV measure}{M} \right)}{S} for L=0$

PWV: Pulse wave velocity
